# Association between life-course cigarette smoking and metabolic syndrome: a discovery-replication strategy

**DOI:** 10.1186/s13098-022-00784-2

**Published:** 2022-01-15

**Authors:** Jingya Wang, Yang Bai, Zihang Zeng, Jun Wang, Ping Wang, Yongai Zhao, Weili Xu, Yun Zhu, Xiuying Qi

**Affiliations:** 1 Department of Epidemiology and Biostatistics, School of Public Health, Tianjin Medical University, Qixiangtai Road 22, Heping district, 300070 Tianjin, People’s Republic of China; 2 Tianjin Key Laboratory of Environment, Nutrition and Public Health, Tianjin, People’s Republic of China; 3 Tianjin Santan Hospital, Nankai District, Tianjin, People’s Republic of China; 4 Aging Research Center, Department of Neurobiology, Care Sciences and Society, Karolinska Institutet, Stockholm, Sweden

**Keywords:** Cigarette smoking, Metabolic syndrome, Discovery-replication strategy, Interaction, Cross-sectional study

## Abstract

**Background:**

The relation between cigarette smoking and metabolic syndrome (MetS) remains unclear, and previous studies focusing on MetS are limited in sample size. We investigated the association between life-course smoking and MetS with independent discovery and replication samples.

**Methods:**

Preliminary analysis utilized data from an annual cross-sectional survey of 15,222 participants aged ≥ 60 years in Tianjin, China. Suggestive associations were followed-up in 8565 adults from the China Health and Nutrition Survey. MetS was identified according to the criteria of the Chinese Diabetes Society in 2013. Life-course smoking was assessed by a comprehensive smoking index (CSI), based on information on smoking intensity, duration, and time since cessation across life-course, collected through standard questionnaires. Participants were divided into four groups: non-smokers; and the tertiles of CSI in ever smokers. Multivariable logistic regression was used to estimate odds ratios (ORs) and 95% confidence intervals (CIs) for the association between life-course smoking and MetS.

**Results:**

In the discovery sample, ORs of MetS were 2.01 (95%CI: 1.64–2.47) and 1.76 (95%CI: 1.44–2.16) for smokers in the highest and second tertile of CSI compared with never smokers. Potential interaction was shown for age, with increased ORs for MetS associated with smoking limited to individuals who aged < 70 years (P_interaction_ = 0.015). We were able to replicate the association between cigarette smoking and MetS in an independent adult sample (second tertile vs. never: OR = 1.30, 95%CI: 1.04–1.63). The interaction of smoking with age was also replicated.

**Conclusions:**

Life-course cigarette smoking is associated with an increased odds of MetS, especially among individuals who aged < 70 years.

**Supplementary Information:**

The online version contains supplementary material available at 10.1186/s13098-022-00784-2.

## Background

Metabolic syndrome (MetS) is a common cluster of cardiovascular risk factors including abdominal obesity, hypertension, hyperglycemia, and dyslipidemia, and is strongly associated with increased risk of diabetes and cardiovascular morbidity [1, 2]. Approximately one quarter of the world’s adult population reportedly have MetS [3]. Exacerbated by modern lifestyles of sedentary behaviors and high-energy-dense diets, the rising trend of MetS has become a major public health problem that would threaten to reverse the health improvements accomplished during the past few decades.

Although the etiology of MetS remains controversial, cigarette smoking has been implicated as an important modifiable risk factor for MetS. Smoking is clearly associated with lipid abnormalities, endothelial dysfunction, and a prothrombotic state [4–7], all of which are components of MetS. These metabolic and hemodynamic abnormalities might also be modulated by the deleterious influence of smoking on insulin resistance [4, 8]. While the positive association between smoking and the presence of MetS has been established in some studies [9–11], these findings could not be confirmed by others [12, 13]. One study conducted among Turkish women has counter-intuitively reported lower risks of MetS among smokers [14].

A limitation in previous studies is that they typically lack a valid and accurate scale of measurement for cumulative smoking exposure. Similar to other long-term exposures to environmental hazards, smoking history is a complex and multi-dimensional phenomenon [15]. To avoid analytical challenges imposed by this multi-dimensionality of exposure, many researchers have adopted a rather simplified analytical approach by focusing on a single aspect of smoking (e.g., smoking status, duration, or intensity); however, ignoring possible influences of other smoking dimensions may cause residual confounding [15]. Although some authors have chosen to simultaneously model several smoking-related variables, this may induce multicollinearity or extremely unstable estimates. While other studies [9, 10] utilized cumulative “pack-years”, calculated as the product of smoking duration and cigarettes per day divided by 20, time since smoking cessation and their complex interplay was not accounted for. Furthermore, the majority of previous studies on smoking and MetS have been limited by sample size [9, 10] and did not replicate their results using similar protocols in independent study populations to assess the robustness of findings.

For all these reasons, we utilized a single aggregate measure to represent all the relevant aspects of smoking history, a rigorous discovery-replication design leveraging data from two independent surveys, as well as large sample sizes to guarantee adequate power. The main research purpose was to discover and replicate the association between life-course tobacco exposure and the MetS thus contributing further to the knowledge gap regarding potential disease etiology.

## Research design and methods

### Study population

We analyzed two independent samples with detailed questionnaire and health examination data available: The Tianjin Nankai Community Health Survey (TNCHS) and the China Health and Nutrition Survey (CHNS, http://www.cpc.unc.edu/projects/china).

The TNCHS is a community-based public-interest program designed to assess and improve the health status of older adults in Tianjin Nankai district. Eligible participants were all older adults aged 60 years and above residing in five communities in Nankai district, Tianjin, China. At enrollment of 2018, 17,219 older adults, accounting for 74% of all eligible participants in the five communities, were recruited and subsequently underwent interviews and health examinations. Exclusions from this analysis were made for individuals who had an equivocal MetS status (n = 1995) and those who lacked information on other crucial covariates (i.e., age, dietary habits, n = 2). As a result, a total of 15,222 participants (7073 men and 8149 women) were included in the final analysis.

The CHNS is a nationwide, household-based, open-cohort survey designed to investigate how the wide-ranging social and economic changes within China have influenced the nutritional and health status of its population [16]. A detailed description regarding the CHNS protocol and the selection methods for a nationwide representative sample of Chinese has been published elsewhere [16]. In brief, initiated with a partial sample in 1989, the full survey ran nine times from 1991 to 2011 in nine Chinese provinces that constituted 47% of China's population [16]. Using a multistage random cluster sampling method in each province, the survey draw a sample of approximately 4,400 households with a total of 19,000 individuals. Sampling weights were calculated based on the 2010 China population census data. As biomarker data were only available in 2009 where participants’ MetS status can be determined, only the CHNS 2009 data were analyzed for the specific purpose of the research. We analyzed adult participants who were aged 18 years or older at the date of questionnaire completion as our replication sample. Exclusions were made if individuals had an equivocal MetS status (n = 41) or provided either an invalid or missing response regarding cigarette smoking (n = 66). As a result, a total of 8565 adults (3976 men and 4,589 women) were included. Written informed consent was obtained from all participants. The survey protocols were approved by the Institutional Review Committees of the University of North Carolina at Chapel Hill, NC, USA, and the China National Institute of Nutrition and Food Safety at the Chinese Center for Disease Control and Prevention, Beijing, China.

### Data collection

The TNCHS survey combined interviews and physical examinations. The interview included questions concerning demographics, socioeconomic status, diet, cigarette smoking, physical activity, and other lifestyle factors. Participants were asked to estimate their average quantity and frequency of alcohol drinking per week during the past year, as well as quantitative dimensions of physical activity including frequency, duration, and types of activities in one week. Dietary habit was measured by self-reported preference for a vegetarian, meat-based, or balanced diet.

The health examination consisted of medical and physiological measurements (e.g., waist circumference, height, and blood pressure), as well as laboratory tests (e.g., fasting plasma glucose, serum triglycerides, high-density lipoprotein (HDL)) administered by highly trained medical personnel using standard protocols. Weight and height were measured without shoes and in light clothing. Body mass index (BMI) was calculated as weight in kilograms divided by the square of height in meters. Waist circumference was measured between the lower rib margin and the anterior superior iliac spine. Measurement of blood pressure was conducted within the medical examination after a resting phase of at least five minutes. Systolic blood pressure and diastolic blood pressure were measured three times on the right brachial artery in the seated position with a regularly tested mercury sphygmomanometer after 5-min rest following a standard protocol. The mean value of the three readings was used as the final blood pressure. Fasting plasma glucose was tested with the glucose oxidase—peroxidase method. HDL levels were assessed using the polyethylene glycol (PEG)-modified enzyme method, while triglyceride levels were measured in serum using the glycerol-3-phosphate oxidase method. All physical examinations were performed in Tianjin Santan hospital.

In each CHNS survey, detailed survey questionnaires were administered in person to gather individual data on demographics, socioeconomics, dietary intake, health history (e.g., medications, key chronic conditions), and health-related behaviors (e.g., cigarette smoking and beverage consumption). Fasting blood samples were taken from all participants in 2009 and were shipped to Beijing Central Laboratory, where all specimens were tested with stringent quality assurance. More detailed information on the CHNS design and biochemical analyses can be found elsewhere [17].

### Smoking history assessment

Participants were asked whether they ever smoked cigarettes in their life. Those responding ‘yes’ were asked about the age at which they started smoking, the number of cigarettes they usually smoked per day, the duration of smoking and, where applicable, the relevant information for when they quitted smoking. In the current study, ever-smoker was defined as a person who declared having smoked for more than one year during their lifetime [9]. A person was considered a current smoker if the person declared still smoking within one year prior to enrollment. If a person who had smoked for at least one year in his/her lifetime but who had quit smoking for more than one year at the time of interview, this person was considered a former smoker.

We applied an aggregate CSI, proposed by Leffondre et al. [15] to measure participants’ life-course smoking exposure. This smoking index incorporated three dimensions of smoking (i.e., smoking intensity (int), duration (dur), and time since cessation (tsc)), and was calculated as follows:

$$ CSI = (1{ - 0}{\text{.5}}^{{{{{\text{dur}}^{*} } \mathord{\left/ {\vphantom {{{\text{dur}}^{*} } {\uptau }}} \right. \kern-\nulldelimiterspace} {\uptau }}}} )(0.5^{{{{tsc^{*} } \mathord{\left/ {\vphantom {{tsc^{*} } {\uptau }}} \right. \kern-\nulldelimiterspace} {\uptau }}}} )\ln ({\text{int}} + 1) $$

where ${\text{tsc}}^{*} = \max (tsc - {{\updelta ; 0)}}$, ${\text{dur}}^{*} = \max (dur + tsc - {{\updelta ; 0}}){\text{ - tsc}}^{*}$. The half-life parameter (τ) and the lag parameter (δ) were fixed a priori at 25 and 0.5 [15].

Taking into account not only the intensity and duration but also the time since quitting smoking makes the CSI a better index to represent the life-course smoking history. Additionally, this aggregate smoking index may avoid collinearity issues and is promising for a good model fit. For this analysis, cigarette smoking was represented by categories of smoking status (never, current, or former), duration of smoking in years (TNCHS: none, > 0- < 39, 39- < 45, ≥ 45; CHNS: none, > 0- < 23, 23- < 35, ≥ 35), number of cigarette daily (none, 1–9, 10–19, and ≥ 20), cigarette pack-years (TNCHS: none, > 0- ≤ 19, > 19- ≤ 36.5, > 36.5; CHNS: none, > 0- < 15, ≥ 15- < 30, ≥ 30), as well as a cumulative CSI score (none, tertiles of CSI score in ever smokers (TNCHS: none, > 0- ≤ 1.550, > 1.550- ≤ 1.940, > 1.940; CHNS: none, > 0- ≤ 1.171, > 1.171- ≤ 1.701, > 1.701)). Study-specific analyses were based on data-dependent tertile cut-points.

### Diagnosis of MetS

The diagnostic criteria for MetS were based on the recommendations from the Chinese Diabetes Society (CDS) in 2013 [1]. MetS patients had to fulfill at least three of the following five criteria: (1) abdominal obesity (waist circumference ≥ 90 cm in males and ≥ 85 cm in females), (2) fasting plasma glucose ≥ 6.1 mmol/L or self-reported drug treatment for elevated glucose, (3) elevated blood pressure (≥ 130/85 mmHg or on drug treatment for hypertension), (4) fasting serum triglycerides ≥ 1.7 mmol/L, and (5) fasting serum HDL < 1.04 mmol/L.

### Statistical analysis

Characteristics of the study population by MetS status were compared using chi-square tests for categorical variables and student's t-test for continuous variables. Multivariable logistic regression models were used to estimate the odds ratios (ORs) and 95% confidence intervals (CIs) for the associations between MetS and categories of exposure, using never smokers as the reference. In the CHNS study, subjects with missing values on any smoking dimensions or covariates (7.1%) were imputed with multiple imputation by the chained equation. In the TNCHS study, subjects with missing values on smoking intensity in former smokers (n = 11) were replaced with the median. In the selection approach of covariates, we evaluated an extensive list of potential confounders, including demographics, socioeconomics, region, diet, alcohol consumption, and other lifestyle factors. The final list of potential confounders included in the model was based on stepwise selection (P_in_ = 0.05, P_out_ = 0.10), including age, physical activity, BMI, and dietary habits in the TNCHS, and sex, age, BMI, province, and region in the CHNS. Potential interactions by demographic and lifestyle characteristics with respect to the smoking-MetS association were assessed in two ways: (1) on a multiplicative scale, evaluated with a Wald test assessing the significance of the interaction terms between CSI categories and the potential effect modifier; and (2) on an additive scale, measured by the relative excess risk due to interaction (RERI), calculated as the difference between the expected risk and the observed risk [18]. Linear trend was tested by considering the median value of each category of smoking variables as a continuous variable in the model.

Since early symptoms of the disease may lead to changes in smoking behavior, in order to reduce bias due to reverse causality, we discounted the three years before the date of interview in computing each smoking variable in the sensitivity analysis. The cut-point of three years was determined based on Leffondré et al.’s recommendations [19]. In addition, to avoid possible inconsistencies caused by different covariates adjusted between samples, we repeated the analysis for the discovery and replication samples while adjusting for the same set of covariates, namely sex, age, and BMI. Supplementary analyses using MetS criteria defined by the 2009 Joint Interim Statement [20], in which the diagnostic threshold values for specific MetS components are slightly different from those in the CDS criteria, were also performed.

The level of statistical significance was set at a P value less than 0.05. All P values were two-tailed. Statistical analyses were performed using SAS software version 9.4 (SAS institute, Cary, NC), Stata SE version 15.0 (Stata Corp, College Station, Texas), and IBM SPSS Statistics 24.0 (IBM Corp, New York, NY).

## Results

### Characteristics of the study populations

Of the 15,222 participants in the TNCHS study, 3546 (23.3%) had MetS. Compared with individuals without MetS, MetS patients were slightly older, more physically active, and more likely to have higher BMI, higher alcohol consumption, and a meat-based diet (Table 1). The prevalence of MetS in the CHNS study was 18.2% based on the 2009 survey. Likewise, MetS patients in the CHNS study were older, consumed more alcohol, and tended to have larger waist circumference and higher BMI relative to participants without MetS. In general, the covariate distribution was relatively comparable between the discovery and replication samples.

**Table 1:** Characteristics of participants by the metabolic syndrome status

Characteristics	Discovery stage: TNCHS study			Replication stage: CHNS study		
	MetS (−) (n = 11,676)	MetS ( +) (n = 3546)	*P* value	MetS (−) (n = 7006)	MetS ( +) (n = 1559)	*P* value
Age (years)	68.81 ± 7.97	70.18 ± 8.10	< 0.001	49.08 ± 15.23	55.47 ± 13.12	< 0.001
Sex			0.195			< 0.001
Female	6217 (53.25)	1,932 (54.48)		3,834 (54.72)	755 (48.34)	
Male	5,459 (46.75)	1,614 (45.52)		3,172 (45.28)	804 (51.57)	
BMI (kg/m^2^)	23.73 ± 2.40	25.92 ± 3.16	< 0.001	22.67 ± 3.10	26.53 ± 3.33	< 0.001
Alcohol consumption			< 0.001			0.037
Never	10,853 (92.95)	3,204 (90.36)		4761 (67.96)	1,038 (66.58)	
Occasional	237 (2.03)	101 (2.85)		816 (11.65)	157 (10.07)	
Often	22 (0.19)	10 (0.28)		788 (11.25)	199 (12.76)	
Every day	515 (4.41)	207 (5.84)		641 (9.15)	165 (10.58)	
Former drinker	49 (0.42)	24 (0.68)		–	–	
Exercise frequency			< 0.001	–	–	–
Never	4743 (40.62)	1212 (34.18)		–	–	
Occasional	171 (1.46)	52 (1.47)		–	–	
Often	805 (6.89)	303 (8.54)		–	–	
Every day	5957 (51.02)	1,979 (55.81)		–	–	
Dietary habits			0.005	–	–	–
Vegetarian diet	109 (0.93)	19 (0.54)		–	–	
Balanced diet	11,508 (98.56)	3497 (98.62)		–	–	
Meat-based diet	59 (0.51)	30 (0.85)		–	–	
Province			–			< 0.001
Liaoning	–	–		572 (8.16)	242 (15.52)	
Heilongjiang	–	–		718 (10.25)	161 (10.33)	
Jiangsu	–	–		890 (12.70)	228 (14.62)	
Shandong	–	–		751 (10.72)	202 (12.96)	
Henan	–	–		772 (11.02)	201 (12.89)	
Hubei	–	–		755 (10.78)	156 (10.01)	
Hunan	–	–		915 (13.06)	169 (10.84)	
Guangxi	–	–		953 (13.60)	105 (6.74)	
Guizhou	–	–		680 (9.71)	95 (6.09)	
Region			–			< 0.001
Urban	–	–		2226 (31.77)	595 (38.17)	
Rural	–	–		4780 (68.23)	964 (61.83)	
Activity likes: sports			–			0.837
Does not participate	–	-		408 (5.82)	87 (5.58)	
Dislike very much	–	–		265 (3.78)	54 (3.46)	
Dislike somewhat	–	–		4187 (59.76)	956 (61.32)	
Neutral	–	–		1,413 (20.17)	307 (19.69)	
Like somewhat	–	–		677 (9.66)	146 (9.36)	
Like very much	–	–		56 (0.80)	9 (0.58)	
Components of MetS						
Abdominal obesity^a^	1534 (13.14)	1943 (54.79)	< 0.001	1542 (22.46)	1283 (82.77)	< 0.001
High blood pressure	3806 (32.60)	2729 (76.96)	< 0.001	2346 (33.49)	1285 (82.42)	< 0.001
High plasma glucose^b^	2546 (21.81)	2478 (69.88)	< 0.001	414 (5.91)	741 (47.53)	< 0.001
High serum TG^a,b^	2650 (23.08)	2438 (70.83)	< 0.001	1308 (18.69)	1318 (84.60)	< 0.001
Low serum HDL^a,b^	1896 (16.64)	2373 (69.16)	< 0.001	411 (5.88)	709 (45.57)	< 0.001

Values are mean ± SD and n (%);

*MetS* metabolic syndrome, *BMI* body mass index, *TG* triglyceride, *HDL* high-density lipoprotein

Abdominal obesity was defined as waist circumference ≥ 90 cm in men and ≥ 85 cm in women; High blood pressure: blood pressure ≥ 130/85 mm Hg or on medication; high plasma glucose: fasting plasma glucose (FPG) ≥ 6.1 mmol/L or on medication; high serum TG: serum TG ≥ 1.7 mmol/L; low serum HDL: serum HDL < 1.04 mmol/L;

^a^Totals may not add up due to missing values in the CHNS study

^b^Totals may not add up due to missing values in the TNCHS study

### Associations between smoking and MetS

Our analysis revealed that in both studies, the prevalence of MetS was higher in current smokers than in the non-smokers (TNCHS: OR = 1.66, 95% CI 1.46–1.89; CHNS: OR = 1.22, 95% CI 1.03–1.45) (Table 2). Notably, current smoking, but not former smoking, was associated with increased OR for MetS, providing further support for the recommendation of smoking cessation. The higher ORs of MetS related to smoking persisted in more detailed definitions of the exposure, including cigarettes daily, smoking duration, pack-years and cumulative smoking exposure classified with the CSI, although, for the latter three variables, the ORs of being in the highest tertile did not quite attain a statistically significant level in the replication.

**Table 2:** Adjusted odds ratios (ORs) and 95% confidence intervals (CIs) of metabolic syndrome associated with cigarette smoking

Cigarette smoking variables	Discovery stage: TNCHS study^a^			Replication stage: CHNS study^b^		
	MetS ( +)/All	*OR* (95%Cl)	*P*_trend_^c^	MetS ( +)/All	*OR* (95%Cl)	*P*_trend_^c^
Non-smoker	3,063/13,739	Reference		1,047/5,977	Reference	–
Smoking status			-			–
Ever-smoker	483/1,483	1.62 (1.43–1.84)		512/2588	1.18 (0.10–1.40)	
Former	28/90	1.11 (0.68–1.80)		46/187	0.85 (0.57–1.27)	
Current	455/1,393	1.66 (1.46–1.89)		466/2401	1.22 (1.03–1.45)	
Intensity of smoking (cig/day)^d^			< 0.001			0.015
1–9	90/357	1.10 (0.85–1.43)		81/427	1.05 (0.77–1.42)	
10–19	186/535	1.73 (1.42–2.11)		131/709	1.09 (0.85–1.40)	
20-	207/591	1.91 (1.58–2.31)		300/1,452	1.27 (1.05–1.54)	
Smoking duration^d^			< 0.001			0.881
Tertile 1	143/471	1.46 (1.17–1.82)		156/846	1.51 (1.18–1.93)	
Tertile 2	154/468	1.72 (1.39–2.14)		197/882	1.41 (1.13–1.76)	
Tertile 3	186/544	1.68 (1.38–2.05)		159/860	0.81 (0.64–1.03)	
Pack-years^d^			< 0.001			0.639
Tertile 1	132/494	1.18 (0.94–1.46)		160/869	1.25 (0.99–1.58)	
Tertile 2	172/494	1.81 (1.47–2.22)		172/830	1.30 (1.04–1.64)	
Tertile 3	179/495	1.97 (1.61–2.42)		180/889	1.03 (0.82–1.29)	
CSI categories^d^			< 0.001			0.364
Tertile 1	132/494	1.19 (0.95–1.48)		161/862	1.30 (1.03–1.65)	
Tertile 2	168/502	1.76 (1.44–2.16)		181/869	1.30 (1.04–1.63)	
Tertile 3	183/487	2.01 (1.64–2.47)		170/857	0.99 (0.78–1.24)	

*CSI* comprehensive smoking index, *OR* odds ratio, *CI* confidence interval

^a^Adjusted for age, physical activity, body mass index, dietary habits

^b^Adjusted for sex, age, body mass index, province, and region

^c^Linear trend was tested by entering the median value of each group of cigarette smoking variables as continuous variables in the models

^d^Participants were divided into four groups: non-smokers; and the tertiles of CSI in ever smokers. In the TNCHS: the tertile cut-off points were: 39 and 45 for years of smoking, 19 and 36.5 for pack-years, and 1.550 and 1.940 for CSI. In the CHNS: the tertiles cut-off points were: 23 and 35 years for years smoking, 15 and 30 for pack-years, and 1.171 and 1.701 for CSI

### Associations between smoking and the components of the MetS

In the discovery stage, when individual MetS components were the outcomes, cumulative tobacco use (i.e., CSI) was independently related to the following components of the syndrome: hypertension (highest tertile vs. never smokers: OR = 2.68, 95% CI 2.19–3.27), hyperglycemia (OR = 1.32, 95% CI 1.09–1.59) and dyslipidemias (decreased HDL: OR = 2.00, 95% CI 1.66–2.41) (Table 3); only the CSI-dyslipidemias association, however, showed reproducibility (decreased HDL: OR = 1.38 (95% CI 1.12–1.71) for the lowest tertile vs. never smokers) (Table 3).

**Table 3:** Adjusted ORs and 95% CIs for the associations between smoking and the components of the metabolic syndrome

Metabolic syndrome components	Discovery stage: TNCHS study^a^					Replication stage: CHNS study^b^				
	Cases/Non-cases	CSI categories			*P*_trend_^c^	Cases/Non-cases	CSI categories			*P*_trend_^c^
		Tertile 1	Tertile 2	Tertile 3			Tertile 1	Tertile 2	Tertile 3	
		*OR* (95%Cl)	*OR* (95%Cl)	*OR* (95%Cl)			*OR* (95%Cl)	*OR* (95%Cl)	*OR* (95%Cl)	
Abdominal obesity	3,477/11,745	0.92 (0.70–1.20)	0.80 (0.61–1.05)	1.12 (0.86–1.47)	0.565	2825/5,740	1.18 (0.93–1.49)	1.11 (0.89–1.40)	0.90 (0.71–1.13)	0.795
High blood pressure	6535/8687	1.97 (1.63–2.39)	2.64 (2.18–3.20)	2.68 (2.19–3.27)	< 0.001	1,155/7410	1.06 (0.88–1.28)	0.92 (0.77–1.10)	0.92 (0.76–1.10)	0.265
High plasma glucose	5,024/10,198	0.91 (0.75–1.11)	1.31 (1.09–1.58)	1.32 (1.09–1.59)	0.001	3,631/4934	0.95 (0.74–1.23)	1.12 (0.89–1.41)	0.81 (0.64–1.02)	0.303
High serum TG	5,088/10,134	0.87 (0.72–1.06)	0.95 (0.78–1.14)	1.04 (0.86–1.26)	0.563	2626/5,939	1.26 (1.05–1.51)	1.26 (1.06–1.50)	0.97 (0.81–1.17)	0.336
Low serum HDL	4269/10,953	1.33 (1.10–1.61)	1.51 (1.25–1.82)	2.00 (1.66–2.41)	< 0.001	1120/7445	1.38 (1.12–1.71)	1.04 (0.83–1.31)	1.05 (0.83–1.33)	0.533

*CSI* comprehensive smoking index, *OR*: odds ratio, *CI* confidence interval, *TG* triglyceride, *HDL* high-density lipoprotein;

^a^Adjusted for age, body mass index, physical activity, dietary habits;

^b^Adjusted for sex, age, body mass index, province, region.;

^c^Linear trend was tested by entering the median value of each group of cigarette smoking variables as continuous variables in the models

### Interactions of smoking with demographic and lifestyle characteristics

The multivariable models were repeated for CSI categories between strata classified by demographic and lifestyle factors (Table 4). Intriguingly, we discovered a significant interaction on a multiplicative scale between CSI and age in MetS (P _interaction_ = 0.015; highest tertile vs. never smoking: OR (95%CI) for individuals aged < 70 years: 2.31 (1.81–2.95); OR (95%CI) for those aged ≥ 70 years: 1.41 (0.95–2.08)). In the replication sample, effect modification of the CSI-MetS association by age was detected on both additive and multiplicative scales. In addition, the elevated ORs associated with smoking were limited to men and those who disliked exercise, although the interaction terms were not statistically significant. Counter-intuitively, the detrimental effect of smoking on MetS appeared to be more pronounced among individuals who never consumed alcohol than past/current alcohol consumers in the TNCHS sample (P _interaction_ = 0.054), but this was not replicated in the CHNS study.

**Table 4:** Multiplicative and additive interactions between CSI and demographic and lifestyle characteristics

Stratified factors	Discovery stage: TNCHS study ^a^							Replication stage: CHNS study ^b^						
	MetS ( +)/All	CSI categories			*P*_trend_^c^	*P*_interaction_^d^	RERI (95%Cl) ^e^	MetS ( +)/All	CSI categories			*P*_trend_^c^	*P*_interaction_^d^	RERI (95%Cl) ^e^
		Tertile 1	Tertile 2	Tertile 3					Tertile 1	Tertile 2	Tertile 3			
		*OR* (95%Cl)	*OR* (95%Cl)	*OR* (95%Cl)					*OR* (95%Cl)	*OR* (95%Cl)	*OR* (95%Cl)			
Sex						0.245	0.31 (− 0.19 to 0.81)						0.312	− 0.07 (− 0.69 to 0.54)
Female	1932/8149	1.19 (0.73–1.96)	1.78 (0.98–3.23)	1.33 (0.67–2.66)	0.050			755/4589	1.03 (0.53–2.01)	1.42 (0.67–3.03)	0.93 (0.45–1.93)	0.738		
Male	1614/7073	1.22 (0.95–1.56)	1.82 (1.46–2.28)	2.15 (1.72–2.69)	< 0.001			804/3976	1.05 (0.80–1.36)	1.26 (1.00–1.60)	1.17 (0.91–1.50)	0.085		
Age						0.015	− 0.35 (− 0.86 to 0.16)						0.001	− 1.29 (− 2.13 to − 0.45)
< 70 years	1988/9544	1.25 (0.96–1.63)	1.87 (1.47–2.37)	2.31 (1.81–2.95)	< 0.001			1313/7591	0.97 (0.76–1.24)	1.38 (1.09–1.76)	1.33 (1.03–1.71)	0.004		
≥ 70 years	1558/5678	1.04 (0.71–1.52)	1.46 (0.99–2.15)	1.41 (0.95–2.08)	0.020			246/974	0.71 (0.29–1.75)	0.61 (0.27–1.37)	1.07 (0.62–1.84)	0.813		
Exercise status						0.542	− 0.10 (− 0.29 to 0.49)						0.930	0.004 (− 0.32 to 0.31)
Exercise/like	2334/9267	1.04 (0.80–1.35)	1.81 (1.42–2.30)	1.97 (1.54–2.53)	< 0.001			462/2608	1.06 (0.72–1.57)	1.28 (0.87–1.90)	0.73 (0.47–1.15)	0.701		
No exercise /dislike	1212/5955	1.68 (1.13–2.50)	1.66 (1.12–2.47)	2.08 (1.43–3.01)	< 0.001			1097/5957	1.44 (1.06–1.95)	1.31 (0.99–1.73)	1.11 (0.84–1.45)	0.180		
Alcohol consumption						0.054	− 0.38 (− 0.83 to 0.07)						0.597	0.16 (− 0.15 to 0.46)
Never consumed alcohol	3204/14,057	1.23 (0.92–1.65)	1.89 (1.43–2.50)	2.48 (1.86–3.30)	< 0.001			1038/5799	1.15 (0.78–1.69)	1.35 (0.95–1.91)	0.88 (0.62–1.24)	0.839		
Past/current consumer	342/1165	1.02 (0.69–1.52)	1.37 (0.94–1.99)	1.44 (1.00–2.08)	0.038			521/2766	1.23 (0.89–1.70)	1.36 (1.00–1.86)	1.31 (0.94–1.82)	0.047		

*CSI* comprehensive smoking index, *OR*: odds ratio, *CI* confidence interval, *BMI* body mass index, *RERI* relative excess risk due to interaction

^a^Adjusted for age, physical activity, BMI, dietary habits

^b^Adjusted for sex, age, BMI, province, region

^c^Linear trend tests were performed by entering the median value of each group of cigarette smoking variables as continuous variables in the models

^d^*P* values for the multiplicative interactions between CSI and stratified factors

^e^In the calculation of RERI, smoking status was modeled as a dichotomous variable (never vs. ever)

Sensitivity analysis discounting three years before index date in computing smoking variables did not alter the overall results (Additional file 1: Tables S1–S3). Similar patterns of results were observed when we adjusted for the same covariates in the analyses for the discovery and replication sets (Additional file 1: Tables S4, S5). Similarly, our findings are consistent when we used the Joint Interim Statement criteria in 2009 to define MetS (data not shown).

## Discussion

This study demonstrated a dose–response relationship between cigarette smoking and the MetS among Chinese adults regardless of what index of exposure was used. The positive association between cumulative smoking exposure and MetS was more pronounced among adults who aged < 70 years. Additionally, we also demonstrated the association between cigarette smoking and decreased HDL in discovery and replication samples. These findings could have significant implications for the future health of the public in light of the still-increasing prevalence of MetS and persistently high smoking rate among Chinese ^7^.

The positive association between smoking and the MetS reported here was in line with a number of studies showing increased rates of the MetS among adult smokers [9, 10, 21, 22]. In a previous meta-analysis of 13 prospective cohort studies[23], active smoking was related to the development of metabolic syndrome, while smoking cessation may reduce the likelihood of metabolic syndrome. However, this study only focused on smoking status and smoking intensity, whereas cumulative smoking exposure was not accounted for. Never before, to the best of our knowledge, has this association been investigated using an aggregate smoking index that simultaneously incorporates multiple smoking-related components (i.e., intensity, duration, and smoking cessation). We observed a 2.01 fold increase in the odds of having MetS among smokers in the highest tertile of CSI relative to never smokers. Additionally, many published articles [4, 9, 24] have reported a stepwise gradient of increased risk for the MetS with increasing smoking intensity and duration, which is similar to our findings. Although one cross-sectional study in China in contrast revealed no relationship between smoking duration, intensity, and MetS [25], that analysis was restricted to female participants and only about 3% of the participants reportedly ever smoked. We suppose that the low prevalence of exposure in that study limited its ability to detect these associations. More importantly, our data demonstrated that the increased risk of MetS in former smokers was no longer significant [9, 11]. Therefore, in current smokers, interventions for smoking cessation are probably among the most cost-effective strategies in MetS management.

The underlying mechanisms of the observed association between cigarette smoking and MetS are not entirely clear. The most widely accepted hypothesis posits a key role of insulin resistance in the etiology of MetS [26]. Smoking may influence MetS through its direct and indirect effects on insulin resistance. Direct effects may be relevant to hormone activation. Specifically, the sympathetic activation induced by smoking might increase circulating levels of insulin-antagonistic hormones, such as catecholamines, cortisol, and growth hormone [27], which might directly reduce insulin sensitivity and subsequently accelerate insulin resistance. Moreover, smoking could increase insulin resistance indirectly through triggering visceral adiposity [28, 29], endothelial dysfunction [30, 31], dysregulation of adipokines [32], endoplasmic reticulum (ER) stress [33], inflammation [34], as well as oxidative stress [35].

We further assessed the relationship between life-course cigarette smoking and the individual components of MetS. Our findings of cigarette smoking being associated with reductions in HDL are consistent with a number of previous studies [4, 9, 10, 36]. A plausible biological basis exists for this association—smoking can alter critical lipid transfer proteins, reducing the activity of lecithin-cholesterol acyl-transferase and altering the activity of cholesterol ester transfer protein and hepatic lipase, which likely promotes a rapid clearance of circulating HDL [37]. In this study, we could not find associations of smoking with abdominal obesity. Epidemiological findings from previous studies on this issue have generally been inconsistent. Some studies [8, 28, 29], but not all [9, 36], reported a positive association between smoking and abdominal obesity. We suppose that the cross-sectional nature of the data impeded our ability to detect these associations. In addition, the positive associations between hypertension, hyperglycemia, and smoking were only observed in the TNCHS study but not in the CHNS survey. This discrepancy is partly due to the differences in age (mean age: 69.1 years in TNCHS and 50.24 years in CHNS) and study region (TNCHS: an urban district of Tianjin; CHNS: rural and urban areas in nine provinces of China) that may influence the differences in smoking and disease prevalence. While the associations of smoking with hypertension and hyperglycemia remain inconclusive in the literature [9, 38], the adverse effects of smoking on cardiovascular diseases could not be ignored and require greater investigation.

A novel aspect of the present study is the inclusion of potential interactions between smoking and other demographic and lifestyle factors. We discovered and successfully replicated a multiplicative interaction between smoking and age in relation to MetS in the two populations. Specifically, the detrimental influence of smoking on MetS was stronger among subjects whose age < 70 years. These differences across age may at least in part be a consequence of a depletion of the susceptibles at older ages, greater exposure misclassification in older adults or, perhaps more importantly, the lower amount smoked by older participants (in this study: mean CSI for participants aged < 70: 0.18; mean CSI for those aged 70 and over: 0.13) [39].

Although the interaction term between smoking and sex was not statistically significant, the association between CSI and MetS was more marked in males, a finding that was consistent with a previous study of 5206 participants in French [21]. The sex difference in MetS etiology may be explained by differences in risk factors between men and women, the influence of female hormones on fat distribution [40], as well as the lower prevalence of smoking in women than in men, making this study underpowered to detect smaller associations among women [23]. Furthermore, the discovery study demonstrated a marginally significant interaction by alcohol drinking in the CSI-MetS association but it was not confirmed in the replication. Since the interaction by alcohol consumption remain unreplicated, we refrain from making assertions about the mechanisms. Future research on interactions between tobacco smoking and other lifestyle behaviors (e.g., alcohol drinking) may facilitate targeted interventions to reduce MetS.

The strengths of this study include the use of a better representation of life-course smoking history and a discovery-replication approach that leveraged two independent, relatively large, and population-based samples of Chinese. The availability of detailed information on personal, demographic, and lifestyle habits also allowed us to assess potential confounders and effect modifiers. Limitations include the possibility of the presence of residual confounding, recall bias, and more importantly, the cross-sectional design of study limiting causal inferences. However, we utilized a more calibrated measurement of life-course tobacco exposure (i.e., CSI) and, in sensitivity analyses, we discounted three years before the date of interview in computing each smoking-related variable to minimize the likelihood of reverse causation. Additionally, it is plausible that a diagnosis of hepatic steatosis may confound or modify the relationship observed between smoking and MetS. However, we are unable to assess this possibility in the study due to the lack of information on participants’ hepatic steatosis status.

In conclusion, this is the first study that we are aware of to illustrate a dose–response, positive association between CSI and the MetS. Smoking cessation in smokers is perhaps one of the most effective options in MetS prevention and control. In particular, individuals who aged < 70 years should be prioritized. Considering that cigarette smoking is the leading preventable cause of death in China, our findings may have profound implications for the future direction of public health policy with respect to tobacco control. Further analyses using biomarkers of tobacco exposure, such as serum cotinine levels and tobacco-specific DNA adducts as quantitative measurements of exposure are warranted.

## Supplementary Information

**Additional file 1.** Additional figures and tables.

## Data Availability

Individual-level data from the China Health and Nutrition Survey are available at http://www.cpc.unc.edu/projects/china. Data from the Tianjin Nankai Community Health Survey are available from the corresponding authors upon reasonable request.

## Abbreviations

TNCHS: The Tianjin Nankai Community Health Survey
CHNS: The China Health and Nutrition Survey
BMI: Body mass index
MetS: Metabolic syndrome
CSI: Comprehensive smoking index
TG: Triglycerides
HDL: High-density lipoprotein
OR: Odds ratio
CI: Confidence interval
RERI: Relative excess risk due to interaction
Int: Smoking intensity
dur: Duration of smoking
tsc: Time since cessation

## Code availability

Custom code for data analysis are available from the corresponding authors upon reasonable request.
