# Supplementary material for: Association between life-course cigarette smoking and metabolic syndrome: a discovery-replication strategy
Source: Diabetol Metab Syndr. 2022 Jan 15;14:11. doi: 10.1186/s13098-022-00784-2 (PMC8761321; doi:10.1186/s13098-022-00784-2)
Supplement: Supplementary file 1 — Additional file 1. Additional figures and tables. [file 13098_2022_784_MOESM1_ESM.docx]

**Association between** **life-****course cigarette smoking and metabolic syndrome: a** **discovery-****replication strategy**

Running title: cigarette smoking and metabolic syndrome

Jingya Wang^a,b†^, *BSC*; Yang Bai^a,b†^, *BSC*; Zihang Zeng^a,b^, *BSC*; Jun Wang^c^, *BSC*; Ping Wang^c^, *BSC*; Yongai Zhao^c^, *BSC*; Weili Xu^a,b,d^, *PhD;* Yun Zhu^a,b^*, *PhD*; Xiuying Qi^a,b^*, *PhD*

^a^ Department of Epidemiology and Biostatistics, School of Public Health, Tianjin Medical University, Tianjin, China

^b^ Tianjin Key Laboratory of Environment, Nutrition and Public Health, Tianjin, China

^c^ Tianjin Santan Hospital, Nankai district, Tianjin, China

^d^ Aging Research Center, Department of Neurobiology, Care Sciences and Society, Karolinska Institutet, Stockholm, Sweden

^†^These authors contributed equally to this manuscript.

*****Co-corresponding authors**

Xiuying Qi, Professor

Dept. of Epidemiology & Biostatistics, School of Public Health

Tianjin Medical University, Tianjin, China

Qixiangtai Road 22, Heping district, 300070, Tianjin, PR, China

Email: [qixiuying@tmu.edu.cn](mailto:qixiuying@tmu.edu.cn)

Yun Zhu, Associate Professor

Dept. of Epidemiology & Biostatistics, School of Public Health

Tianjin Medical University, Tianjin, China

Qixiangtai Road 22, Heping district, 300070, Tianjin, PR, China

Email: [yun.zhu@tmu.edu.cn](mailto:yun.zhu@tmu.edu.cn)

**Online-only Supplement Material**

**Table S1.** Adjusted odds ratios (ORs) and 95% confidence intervals (CIs) of metabolic syndrome associated with cigarette smoking

| Cigarette smoking variables | Discovery stage: TNCHS study ^a^ | | | Replication stage: CHNS study ^b^ | | |
| --- | --- | --- | --- | --- | --- | --- |
|  | MetS(+)/All | *OR* (95%Cl) | *P* _trend_ ^c^ | MetS(+)/All | *OR* (95%Cl) | *P* _trend_ ^c^ |
| Non-smoker | 3,063/13,739 | Reference |  | 1,053/6,051 | Reference |  |
| Smoking status |  |  | — |  |  | — |
| Ever-smoker | 483/1,483 | 1.63(1.43-1.85) |  | 506/2,514 | 1.19(1.00-1.40) |  |
| Former | 28/90 | 0.90(0.42-1.96) |  | 33/114 | 0.95(0.59-1.53) |  |
| Current | 455/1,393 | 1.65(1.45-1.88) |  | 473/2,400 | 1.20(1.01-1.42) |  |
| Intensity of smoking(cig/day) ^d^ |  |  | <0.001 |  |  | 0.016 |
| 1-9 | 90/357 | 1.07(0.82-1.39) |  | 78/390 | 1.06(0.78-1.44) |  |
| 10-19 | 186/535 | 1.74(1.42-2.12) |  | 130/691 | 1.11(0.86-1.42) |  |
| 20- | 207/591 | 1.94(1.60-2.34) |  | 298/1,433 | 1.27(1.05-1.54) |  |
| Smoking duration ^d^ |  |  | <0.001 |  |  | 0.712 |
| Tertile 1 | 143/471 | 1.57(1.27-1.94) |  | 166/832 | 1.61(1.26-2.05) |  |
| Tertile 2 | 154/468 | 1.74(1.41-2.15) |  | 177/804 | 1.37(1.09-1.72) |  |
| Tertile 3 | 186/544 | 1.58(1.28-1.95 |  | 163/878 | 0.81(0.64-1.02) |  |
| Pack-years ^d^ |  |  | <0.001 |  |  | 0.838 |
| Tertile 1 | 132/494 | 1.28(1.03-1.58) |  | 163/851 | 1.25(0.99-1.58) |  |
| Tertile 2 | 172/494 | 1.70(1.39-2.09) |  | 173/806 | 1.37(1.09-1.73) |  |
| Tertile 3 | 179/495 | 1.98(1.60-2.44) |  | 170/857 | 0.98(0.78-1.24) |  |
| CSI categories ^d^ |  |  | <0.001 |  |  | 0.544 |
| Tertile 1 | 132/494 | 1.30(1.05-1.61) |  | 167/838 | 1.39(1.10-1.75) |  |
| Tertile 2 | 168/502 | 1.70(1.38-2.10) |  | 176/841 | 1.29(1.03-1.62) |  |
| Tertile 3 | 183/487 | 1.94(1.58-2.38) |  | 163/835 | 0.94(0.75-1.19) |  |

Abbreviations: CSI: comprehensive smoking index; OR: odds ratio; CI: confidence interval;

^a^ Adjusted for age, body mass index, physical activity, dietary habits;

^b^ Adjusted for sex, age, body mass index, province, region;

^c^ Linear trend was tested by entering the median value of each group of cigarette smoking variables as continuous variables in the models;

^d^ Participants were divided into four groups: non-smokers; and tertiles of CSI in ever smokers. In the TSHES: the tertiles cut-off points of were: 37 and 43 for years of smoking, 18 and 34 for pack-years, 1.502 and 1.870 for CSI. In the CHNS : the tertiles cut-off points were: 21 and 32 for years of smoking, 14 and 28 for pack years, 1.100 and 1.625 for CSI.

**Table S2.** Adjusted ORs and 95% CIs for the associations between smoking and the components of the metabolic syndrome

| Metabolic syndrome components | Discovery stage: TNCHS study ^a^ | | | | | Replication stage: CHNS study ^b^ | | | | |
| --- | --- | --- | --- | --- | --- | --- | --- | --- | --- | --- |
|  | Cases/Non-cases | CSI categories | | | *P* _trend_ ^c^ | Cases/Non-cases | CSI categories | | | *P* _trend_ ^c^ |
|  |  | Tertile 1 | Tertile 2 | Tertile 3 |  |  | Tertile 1 | Tertile 2 | Tertile 3 |  |
|  |  | *OR*(95%Cl) | *OR*(95%Cl) | *OR*(95%Cl) |  |  | *OR*(95%Cl) | *OR*(95%Cl) | *OR*(95%Cl) |  |
| Abdominal obesity | 3,477/11,745 | 0.95(0.73-1.24) | 0.81(0.61-1.07) | 1.12(0.86-1.45) | 0.674 | 2,825/5,740 | 1.22(0.96-1.54) | 1.10(0.88-1.38) | 0.89(0.70-1.12) | 0.728 |
| High blood pressure | 6,535/8,687 | 2.04(1.68-2.46) | 2.68(2.19-3.28) | 2.64(2.17-3.21) | <0.001 | 1,155/7,410 | 1.05(0.87-1.26) | 0.95(0.79-1.14) | 0.88(0.74-1.06) | 0.205 |
| High plasma glucose | 5,024/10,198 | 0.98(0.81-1.19) | 1.26(1.04-1.53) | 1.29(1.07-1.56) | 0.002 | 3,631/4,934 | 1.05(0.81-1.34) | 1.11(0.89-1.40) | 0.79(0.62-1.00) | 0.233 |
| High serum TG | 5,088/10,134 | 0.85(0.70-1.03) | 0.98(0.80-1.19) | 1.02(0.85-1.24) | 0.547 | 2,626/5,939 | 1.30(1.09-1.56) | 1.25(1.05-1.50) | 0.94(0.78-1.13) | 0.499 |
| Low serum HDL | 4,269/10,953 | 1.40(1.15-1.69) | 1.44(1.19-1.75) | 1.97(1.64-2.38) | <0.001 | 1,120/7,445 | 1.38(1.12-1.71) | 0.99(0.79-1.25) | 0.99(0.78-1.26) | 0.890 |

Abbreviations: CSI: comprehensive smoking index.; OR: odds ratio; CI: confidence interval; TG, triglyceride; HDL, high-density lipoprotein;

^a^ Adjusted for age, body mass index, physical activity, dietary habits;

^b^ Adjusted for sex, age, body mass index, province, region;

^c^ Linear trend was tested by entering the median value of each group of cigarette smoking variables as continuous variables in the models.

**Table S3.** Multiplicative and additive interactions between CSI and demographic and lifestyle characteristics

| Stratified factors | Discovery stage: TNCHS study ^a^ | | | | | | | Replication stage: CHNS study ^b^ | | | | | | |
| --- | --- | --- | --- | --- | --- | --- | --- | --- | --- | --- | --- | --- | --- | --- |
|  | MetS(+)/All | CSI categories | | | *P*_trend_ ^c^ | *P* _Interaction_ ^d^ | RERI (95%Cl) ^e^ | MetS(+)/All | CSI categories | | | *P*_trend_ ^c^ | *P* _Interaction_ ^d^ | RERI (95%Cl) ^e^ |
|  |  | Tertile 1 | Tertile 2 | Tertile 3 |  |  |  |  | Tertile 1 | Tertile 2 | Tertile 3 |  |  |  |
|  |  | *OR*(95%Cl) | *OR*(95%Cl) | *OR*(95%Cl) |  |  |  |  | *OR*(95%Cl) | *OR*(95%Cl) | *OR*(95%Cl) |  |  |  |
| Sex |  |  |  |  |  | 0.199 | 0.41 (-0.08 to 0.90) |  |  |  |  |  | 0.153 | -0.16 (-0.81 to 0.49) |
| Female | 1,932/8,149 | 1.03(0.61-1.75) | 1.86(1.00-3.46) | 1.28(0.65-2.54) | 0.011 |  |  | 755/4,589 | 1.19(0.61-2.35) | 1.20(0.55-2.60) | 1.10(0.53-2.27) | 0.564 |  |  |
| Male | 1,614/7,073 | 1.40(1.10-1.78) | 1.75(1.38-2.21) | 2.08(1.66-2.60) | <0.001 |  |  | 804/3,976 | 1.14(0.88-1.47) | 1.28(1.00-1.63) | 1.11(0.86-1.44) | 0.133 |  |  |
| Age |  |  |  |  |  | 0.013 | -0.40 (-0.90 to 0.11) |  |  |  |  |  | <0.001 | -1.32 (-2.17 to -0.47) |
| <70 years | 1,988/9,544 | 1.43(1.11-1.85) | 1.78(1.38-2.29) | 2.25(1.76-2.87) | <0.001 |  |  | 1,313/7,591 | 1.07(0.84-1.36) | 1.44(1.13-1.83) | 1.32(1.03-1.71) | 0.003 |  |  |
| ≥70 years | 1,558/5,678 | 1.01(0.68-1.51) | 1.46(0.99-2.16) | 1.34(0.91-1.98) | 0.033 |  |  | 246/974 | 0.81(0.30-2.20) | 0.55(0.24-1.24) | 1.09(0.64-1.86) | 0.828 |  |  |
| Exercise status |  |  |  |  |  | 0.684 | 0.06 (-0.33 to 0.45) |  |  |  |  |  | 0.901 | -0.007 (-0.31 to 0.32) |
| Exercise/like | 2,334/9,267 | 1.16(0.90-1.50) | 1.76(1.37-2.26) | 1.92(1.50-2.46) | <0.001 |  |  | 462/2,608 | 1.52(1.13-2.05) | 1.39(1.05-1.83) | 1.04(0.79-1.37) | 0.253 |  |  |
| No exercise /dislike | 1,212/5,955 | 1.76(1.19-2.62) | 1.57(1.04-2.37) | 1.97(1.36-2.85) | <0.001 |  |  | 1,097/5,957 | 1.16(0.79-1.70) | 1.11(0.74-1.64) | 0.75(0.48-1.18) | 0.533 |  |  |
| Alcohol consumption |  |  |  |  |  | 0.034 | -0.33 (-0.78 to 0.12) |  |  |  |  |  | 0.760 | 0.13 (-0.18 to 0.44) |
| Never consumed alcohol | 3,204/14,057 | 1.26(0.95-1.68) | 1.88(1.40-2.51) | 2.39(1.80-3.19) | <0.001 |  |  | 1,038/5,799 | 1.32(0.90-1.93) | 1.26(0.88-1.80) | 0.89(0.63-1.25) | 0.903 |  |  |
| Past/current consumer | 342/1,165 | 1.23(0.84-1.81) | 1.30(0.89-1.91) | 1.39(0.97-2.00) | 0.053 |  |  | 521/2,766 | 1.29(0.94-1.77) | 1.39(1.02-1.90) | 1.22(0.87-1.72) | 0.079 |  |  |

Abbreviations: CSI: comprehensive smoking index.; OR: odds ratio; CI: confidence interval; BMI, body mass index; RERI: the relative excess risk due to interaction;

^a^ Adjusted for age, physical activity, BMI, dietary habits;

^b^ Adjusted for sex, age, BMI, province, region;

^c^ Linear trend tests were performed by entering the median value of each group of cigarette smoking variables as continuous variables in the models;

^d^ *P* values for the multiplicative interactions between CSI and stratified factors;

^e^ In the calculation of RERI, smoking status was modeled as a dichotomous variable (never vs. ever).

**Table S4.** Adjusted odds ratios (ORs) and 95% confidence intervals (CIs) of metabolic syndrome associated with cigarette smoking

| Cigarette smoking variables | Discovery stage: TNCHS study ^a^ | | | Replication stage: CHNS study ^a^ | | |
| --- | --- | --- | --- | --- | --- | --- |
|  | MetS(+)/All | *OR* (95%Cl) | *P* _trend_ ^b^ | MetS(+)/All | *OR* (95%Cl) | *P* _trend_ ^b^ |
| Non-smoker | 3,063/13,739 | Reference |  | 1,047/5,977 | Reference |  |
| Smoking status |  |  | - |  |  | - |
| Ever-smoker | 483/1,483 | 1.70(1.49-1.94) |  | 512/2,588 | 1.18(0.10-1.40) |  |
| Former | 28/90 | 1.18(0.73-1.91) |  | 46/187 | 0.89(0.60-1.32) |  |
| Current | 455/1,393 | 1.75(1.53-2.00) |  | 466/2,401 | 1.22(1.03-1.44) |  |
| Intensity of smoking(cig/day) ^c^ |  |  | <0.001 |  |  | 0.014 |
| 1-9 | 90/357 | 1.16(0.89-1.50) |  | 81/427 | 1.04(0.77-1.41) |  |
| 10-19 | 186/535 | 1.83(1.50-2.24) |  | 131/709 | 1.11(0.87-1.42) |  |
| 20- | 207/591 | 2.00(1.65-2.43) |  | 300/1,452 | 1.27(1.05-1.54) |  |
| Smoking duration ^c^ |  |  | <0.001 |  |  | 0.839 |
| Tertile 1 | 143/471 | 1.50(1.20-1.87) |  | 156/846 | 1.53(1.20-1.95) |  |
| Tertile 2 | 154/468 | 1.83(1.47-2.28) |  | 197/882 | 1.42(1.14-1.77) |  |
| Tertile 3 | 186/544 | 1.78(1.46-2.18) |  | 159/860 | 0.81(0.64-1.02) |  |
| Pack-years ^c^ |  |  | <0.001 |  |  | 0.621 |
| Tertile 1 | 132/494 | 1.23(0.99-1.54) |  | 160/869 | 1.26(1.00-1.59) |  |
| Tertile 2 | 172/494 | 1.91(1.55-2.36) |  | 172/830 | 1.28(1.02-1.61) |  |
| Tertile 3 | 179/495 | 2.08(1.68-2.56) |  | 180/889 | 1.04(0.83-1.30) |  |
| CSI categories ^c^ |  |  | <0.001 |  |  | 0.367 |
| Tertile 1 | 132/494 | 1.24(1.00-1.55) |  | 161/862 | 1.32(1.04-1.67) |  |
| Tertile 2 | 168/502 | 1.86(1.51-2.29) |  | 181/869 | 1.28(1.02-1.60) |  |
| Tertile 3 | 183/487 | 2.12(1.72-2.61) |  | 170/857 | 1.00(0.79-1.25) |  |

Abbreviations: CSI: comprehensive smoking index; OR: odds ratio; CI: confidence interval;

^a^ Adjusted for age, gender, body mass index;

^b^ Linear trend was tested by entering the median value of each group of cigarette smoking variables as continuous variables in the models;

^c^ Participants were divided into four groups: non-smokers; and tertiles of CSI in ever smokers. In the TNCHS: the tertile cut-off points were: 39 and 45 for years of smoking, 19 and 36.5 for pack-years, and 1.550 and 1.940 for CSI. In the CHNS : the tertiles cut-off points were: 23 and 35 years for years smoking, 15 and 30 for pack-years, and 1.171 and 1.701 for CSI.

**Table S5.** Adjusted ORs and 95% CIs for the associations between smoking and the components of the metabolic syndrome

| Metabolic syndrome components | Discovery stage: TNCHS study ^a^ | | | | | Replication stage: CHNS study ^a^ | | | | |
| --- | --- | --- | --- | --- | --- | --- | --- | --- | --- | --- |
|  | Cases/Non-cases | CSI categories | | | *P* _trend_ ^b^ | Cases/Non-cases | CSI categories | | | *P* _trend_ ^b^ |
|  |  | Tertile 1 | Tertile 2 | Tertile 3 |  |  | Tertile 1 | Tertile 2 | Tertile 3 |  |
|  |  | *OR*(95%Cl) | *OR*(95%Cl) | *OR*(95%Cl) |  |  | *OR*(95%Cl) | *OR*(95%Cl) | *OR*(95%Cl) |  |
| Abdominal obesity | 3,477/11,745 | 1.14(0.87-1.49) | 1.06(0.80-1.39) | 1.46(1.11-1.91) | 0.017 | 2,825/5,740 | 1.20(0.95-1.51) | 1.11(0.89-1.39) | 0.89(0.71-1.12) | 0.762 |
| High blood pressure | 6,535/8,687 | 2.38(1.96-2.88) | 3.26(2.68-3.96) | 3.27(2.68-4.00) | <0.001 | 1,155/7,410 | 1.06(0.88-1.28) | 0.92(0.77-1.09) | 0.92(0.77-1.10) | 0.268 |
| High plasma glucose | 5,024/10,198 | 0.89(0.73-1.08) | 1.28(1.06-1.54) | 1.28(1.06-1.55) | 0.007 | 3,631/4,934 | 0.98(0.76-1.26) | 1.12(0.89-1.40) | 0.82(0.65-1.03) | 0.334 |
| High serum TG | 5,088/10,134 | 0.88(0.72-1.07) | 0.96(0.79-1.16) | 1.05(0.87-1.28) | 0.726 | 2,626/5,939 | 1.25(1.04-1.49) | 1.26(1.06-1.51) | 0.99(0.83-1.20) | 0.254 |
| Low serum HDL | 4,269/10,953 | 1.16(0.95-1.41) | 1.28(1.06-1.55) | 1.69(1.40-2.04) | <0.001 | 1,120/7,445 | 1.37(1.11-1.69) | 1.02(0.81-1.28) | 1.05(0.83-1.33) | 0.630 |

Abbreviations: CSI: comprehensive smoking index.; OR: odds ratio; CI: confidence interval; TG, triglyceride; HDL, high-density lipoprotein;

^a^ Adjusted for age, gender, body mass index;

^b^ Linear trend was tested by entering the median value of each group of cigarette smoking variables as continuous variables in the models.
